# Supplementary material for: The integration of large-scale public data and network analysis uncovers molecular characteristics of psoriasis
Source: Hum Genomics. 2022 Nov 28;16:62. doi: 10.1186/s40246-022-00431-x (PMC9703794; doi:10.1186/s40246-022-00431-x)
Supplement: Supplementary file 1 — Additional file 1. Additional material including supplemetary figures and tables. [file 40246_2022_431_MOESM1_ESM.docx]

| **GEO ID** | **Title** | **PMID** |
| --- | --- | --- |
| GSE102725 | PSORITUS - Secukinumab study in PSOriasis exploring pruRITUS intensity and lesional biomarkers (CAIN457ADE03) | Unpublished |
| GSE106992 | Cellular and Molecular Changes in Psoriasis Lesions Induced by Ustekinumab: Distinct Differences in Responders vs. Non-Responders | 30703387 |
| GSE117239 | Cellular and Molecular Changes in Psoriasis Lesions Induced by Ustekinumab: Distinct Differences in Responders vs. Non responders | 30703387 |
| GSE11903 | Effective treatment of psoriasis with etanercept is linked to suppression of IL17 signaling, not immediate response TNF | 19895991; 27667537 |
| GSE13355 | Gene expression data of skin from psoriatic patients and normal controls | 19169254; 21483750; 21129726 |
| GSE14905 | Type I Interferon: Potential Therapeutic Target for Psoriasis? | 18648529 |
| GSE26866 | Combined Use of Laser Capture Microdissection and Microarray Analysis Identifies Locally Expressed Disease-Related Genes in Focal Regions of Psoriasis Vulgaris Skin Lesions | 22402443 |
| GSE2737_GPL91 | Affected and unaffected skin of 4 psoriatic patients and normal skin of 3 psoriasis free individuals | 16283139 |
| GSE30768 | Post-therapeutic relapse of psoriasis associated with CD11a blockade is associated with T cells and inflammatory myeloid DCs | 22348003 |
| GSE30999 | Expression data from skin biopsy samples from patients with moderate-to-severe psoriasis | 22763790; 27667537 |
| GSE34248 | Gene expression profiling in psoriatic lesional and non-lesional skin [Set 1] | 23308107 |
| GSE41662 | Gene expression profiling in psoriatic lesional and non-lesional skin [Set 2] | 23308107 |
| GSE41663 | Re-analysis by microarray using cDNA target of samples from psoriasis patients enrolled in an etanercept trial | 23308107 |
| GSE41664 | Comparison of Gene Expression in Psoriatic Skin from Different Sources | 23308107 |
| GSE47751 | Early tissue responses to etanercept in psoriasis lesions | 24601997 |
| GSE50790 | Gene expression in psoriasis lesions and uninvolved skin | 22479649 |
| GSE51440 | Guselkumab (interleukin-23-specific monoclonal antibody) demonstrates clinical and molecular response in moderate-to-severe psoriasis | 24679469 |
| GSE53552 | Gene expression profiling in psoriatic lesional and non-lesional skin [brodalumab treatment] | 24646743 |
| GSE57225 | Intra-individual genome expression analysis reveals a specific molecular signature of psoriasis and eczema | 25009230 |
| GSE57376 | Synovial biopsies from RA and PsA patients and skin biopsies from Psoriasis patients under Infliximab treatment | 25333715 |
| GSE67853 | Exploring Molecular Determinants of Disease Progression in Psoriasis by Comparing Different Clinical Subtypes Having Similar Core Transcriptomes | 26763436 |
| GSE69967 | Pathologic Immune Pathways in Psoriasis are Rapidly Attenuated by Tofacitinib Treatment: A Randomized Phase 2 Study in Patients with Moderate to Severe Psoriasis | 27059729; 27667537 |
| GSE75343 | Molecular and cellular profiling of scalp psoriasis reveals differences and similarities compared to skin psoriasis | 26849645 |

*Table S1 - Transcriptomics datasets utilized in the integrated gene expression analysis.*

| **IPKB column name** | **Category** | **Data type** | **Source** | **Description** | **PMID** | **Range** |
| --- | --- | --- | --- | --- | --- | --- |
| ensembl_gene_id | General | text | Ensembl | Ensembl Gene ID | NA | NA |
| hgnc_symbol | General | text | Ensembl | HGNC symbol | NA | NA |
| Uniprot_Accessions | General | list | Ensembl | Uniprot ID | NA | NA |
| Gene_Description | General | text | Ensembl | Description | NA | NA |
| Aliases | General | list | Ensembl | Gene alias | NA | NA |
| ImmunePathwayCytokineSignaling | Immune Pathways / Modules | YN | Reactome | NA | NA | Y/N |
| ImmunePathwayInnate | Immune Pathways / Modules | YN | Reactome | NA | NA | Y/N |
| ImmunePathwayAdaptive | Immune Pathways / Modules | YN | Reactome | NA | NA | Y/N |
| DruggabilitySmallMolecule | Druggability / Tractability | YN | Druggable Genome | NA | 28356508 | Y/N |
| DruggabilityBiologics | Druggability / Tractability | YN | Druggable Genome | NA | 28356508 | Y/N |
| TractabilitySmallMolecule | Druggability / Tractability | YN | https://docs.targetvalidation.org/getting-started/target-tractability | NA | NA | Y/N |
| TractabilityAntibody | Druggability / Tractability | YN | https://docs.targetvalidation.org/getting-started/target-tractability | NA | NA | Y/N |
| Pharos | Druggability / Tractability | YN | https://pharos.nih.gov/idg/index | NA | NA | Y/N |
| MembranomePlosOne | Druggability / Tractability | YN | https://www.proteinatlas.org/humanproteome/tissue/secretome | NA | 20668533 | Y/N |
| MembraneProteinAtlas | Druggability / Tractability | YN | Membrane Protein Atlas | NA | NA | Y/N |
| SecretoryProteinAtlas | Druggability / Tractability | YN | https://www.proteinatlas.org/humanproteome/tissue/secretome | NA | NA | Y/N |
| HumanKOPakistan | Human KO / Trial | YN | PMID: 28406212 | NA | 28406212 | Y/N |
| HumanKOBritishPakistani | Human KO / Trial | YN | PMID: 26940866 | NA | 26940866 | Y/N |
| ClinicalTrial | Human KO / Trial | YN | Metabase; Open Targets | Gene under clinical trial | NA | Y/N |
| uniprotswissprot | General | text | Ensembl | Uniprot/SwissProt ID | NA | NA |
| entrezgene | General | text | Ensembl | Entrez gene ID | NA | NA |
| chromosome_name | Gene Annotation | text | Ensembl | Chromosome | NA | 1 to 22, X, Y, MT |
| degree_lesional | Network_derived | numeric | Experimental_derived | Degree in the lesional network | NA | 1 to 1178 |
| betweenness_lesional | Network_derived | numeric | Experimental_derived | Betweenness in the lesional network | NA | 0 to 88925.4 |
| closeness_lesional | Network_derived | numeric | Experimental_derived | Closeness in the lesional network | NA | 4.119e-05 to 7.373e-05 |
| cc | Network_derived | numeric | Experimental_derived | Clustering coefficient in the lesional network | NA | 0.303 to 0.303 |
| degree_nonlesional | Network_derived | numeric | Experimental_derived | Degree in the non-lesional network | NA | 1 to 1887 |
| betweenness_nonlesional | Network_derived | numeric | Experimental_derived | Betweenness in the non-lesional network | NA | 0 to 51367 |
| closeness_nonlesional | Network_derived | numeric | Experimental_derived | Closeness in the non-lesional network | NA | 4.455e-05 to 7.849e-05 |
| cc_nl | Network_derived | numeric | Experimental_derived | Clustering coefficient in the non-lesional network | NA | 0.3153 to 0.3153 |
| IsBridge | Network_derived | YN | Experimental_derived | Bridge gene | NA | Y/N |
| Bridge_shortpath | Network_derived | numeric | Experimental_derived | Number of connected couples of DEGs | NA | 1 to 696 |
| INfORMrank | Network_derived | numeric | Experimental_derived | Lesional network rank | NA | 1 to 7310 |
| Module_lesional | Network_derived | numeric | Experimental_derived | Module in the lesional network | NA | 1 to 13 |
| Module_nonlesional | Network_derived | numeric | Experimental_derived | Module in the non-lesional network | NA | 1 to 10 |
| Pso_GWAS_p1e-5 | Genetic_association | YN | PMID: 30445434 | p-value < 1e-5 | 30445434 | Y or N |
| Pso_GWAS_p5e-8 | Genetic_association | YN | PMID: 30445434 | p-value < 5e-8 | 30445434 | Y or N |
| Pso_Open_Targets | Genetic_association | YN | https://docs.targetvalidation.org | Genetic association score >= 0.1 | NA | Y or N |

*Table S2 - Integrated Psoriasis Knowledge Base (IPKB) columns description.*


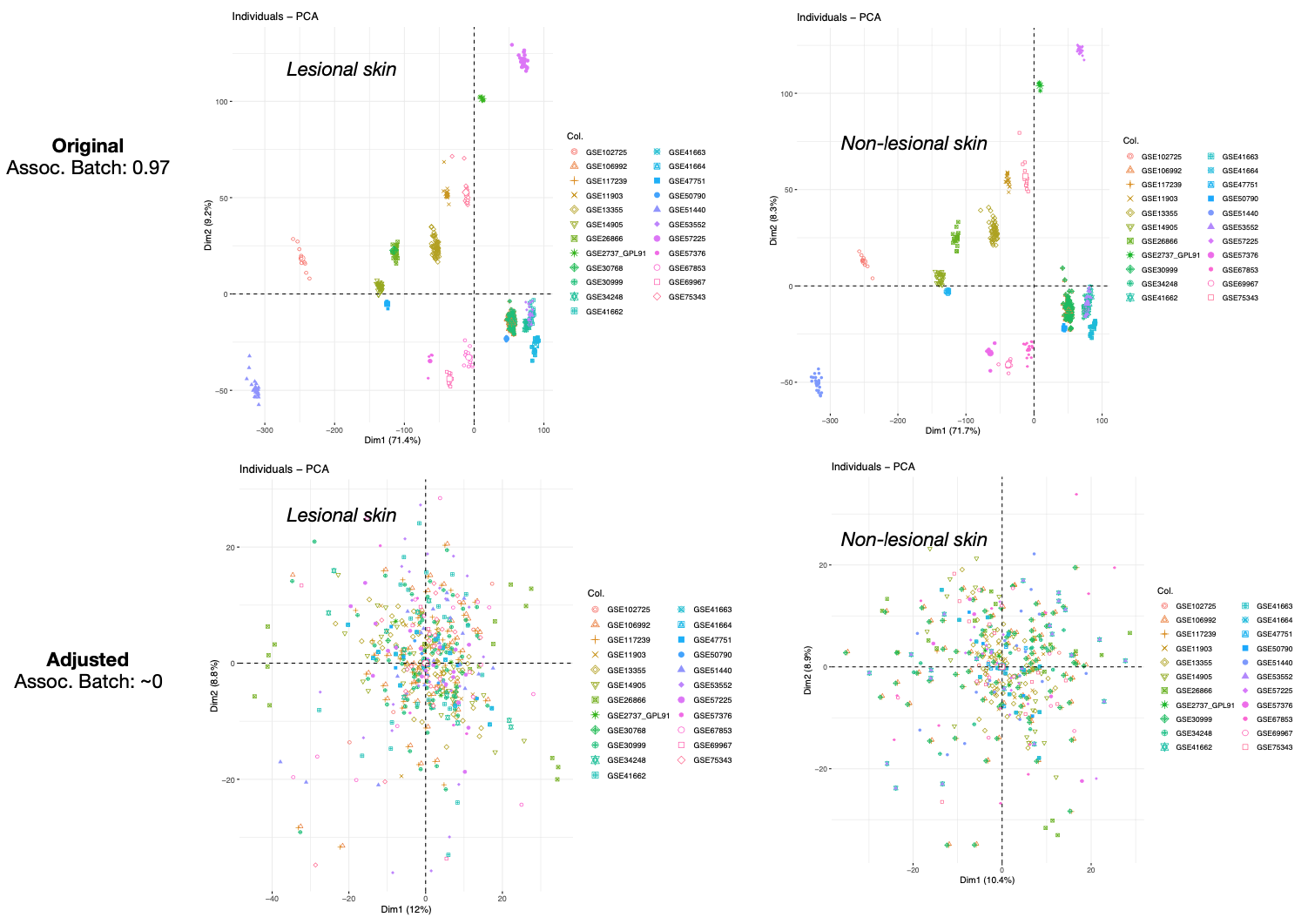


*Figure S1 – Effects of gene expression adjustment to mitigate the batch effect arising from the integration of data deriving from different experiments.*


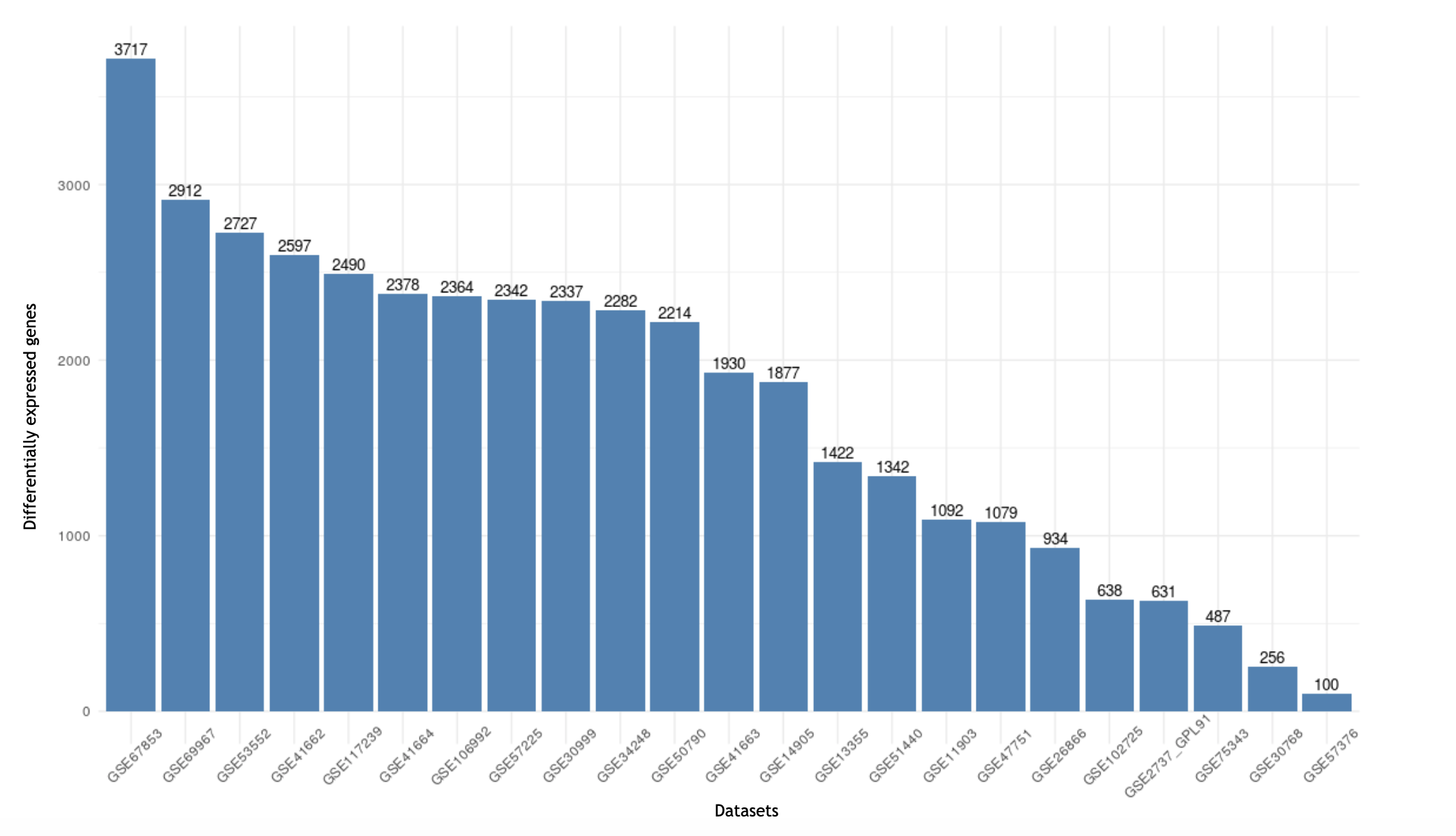


*Figure S2 - The barplot shows the number of differentially expressed genes detected in each dataset included in the meta-analysis.*

*
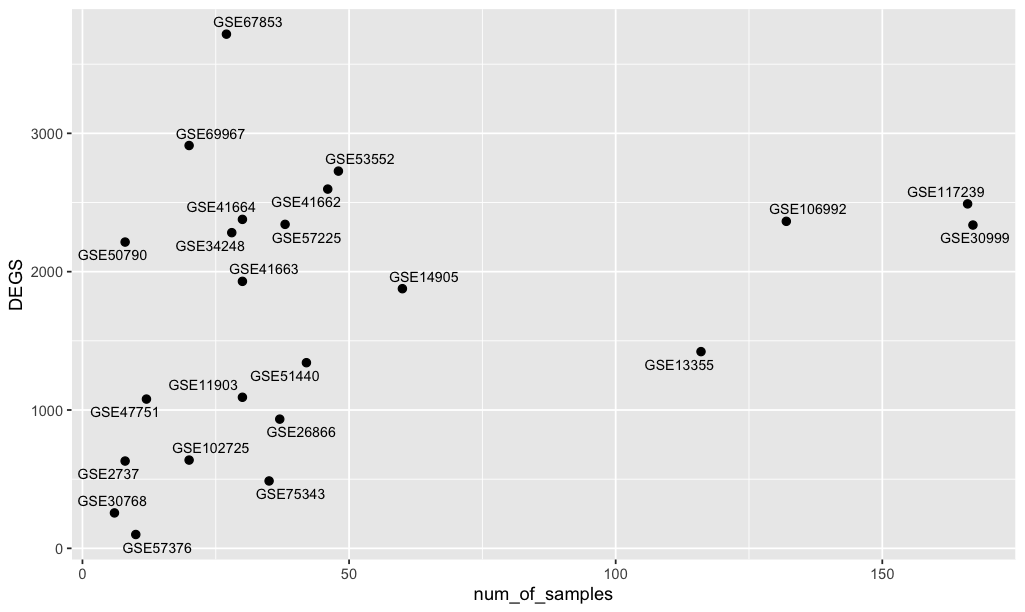
*

*Figure S3 – Relationship between the number of differentially expressed genes and the sample sizes of the analysed datasets.*


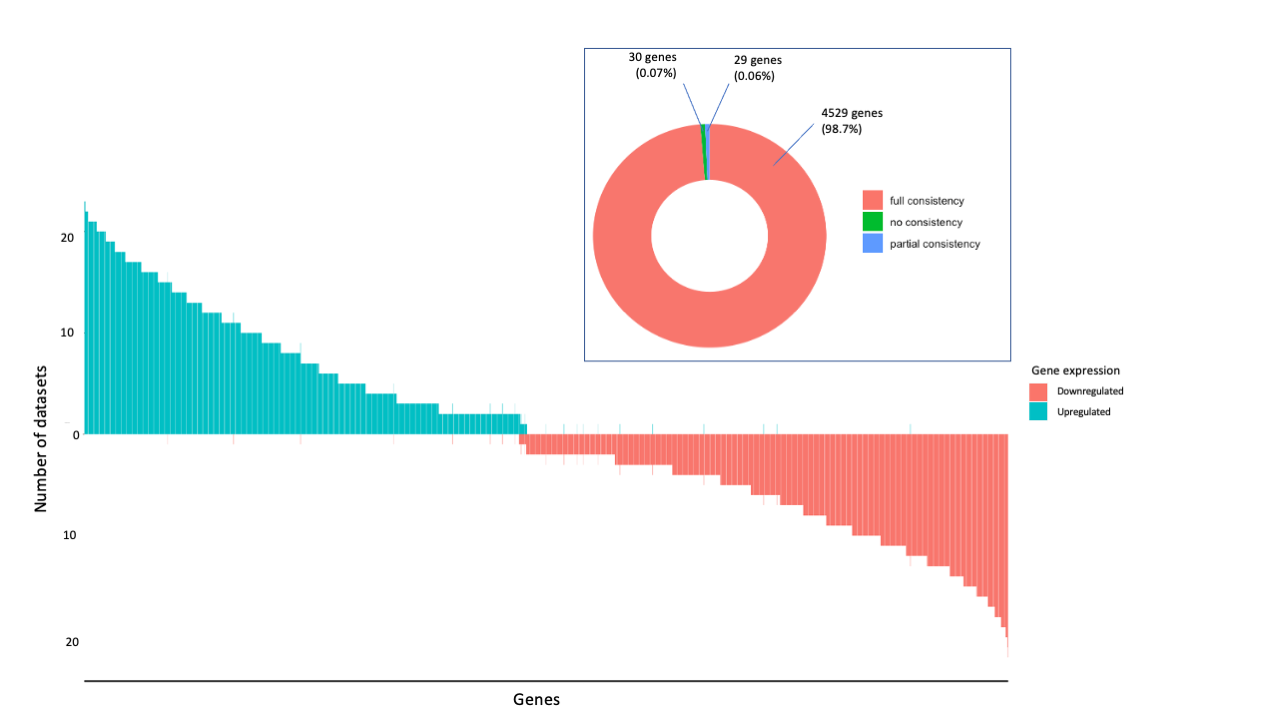


*Figure S4 – Consistency of gene expression trend of differentially expressed genes across the datasets.*


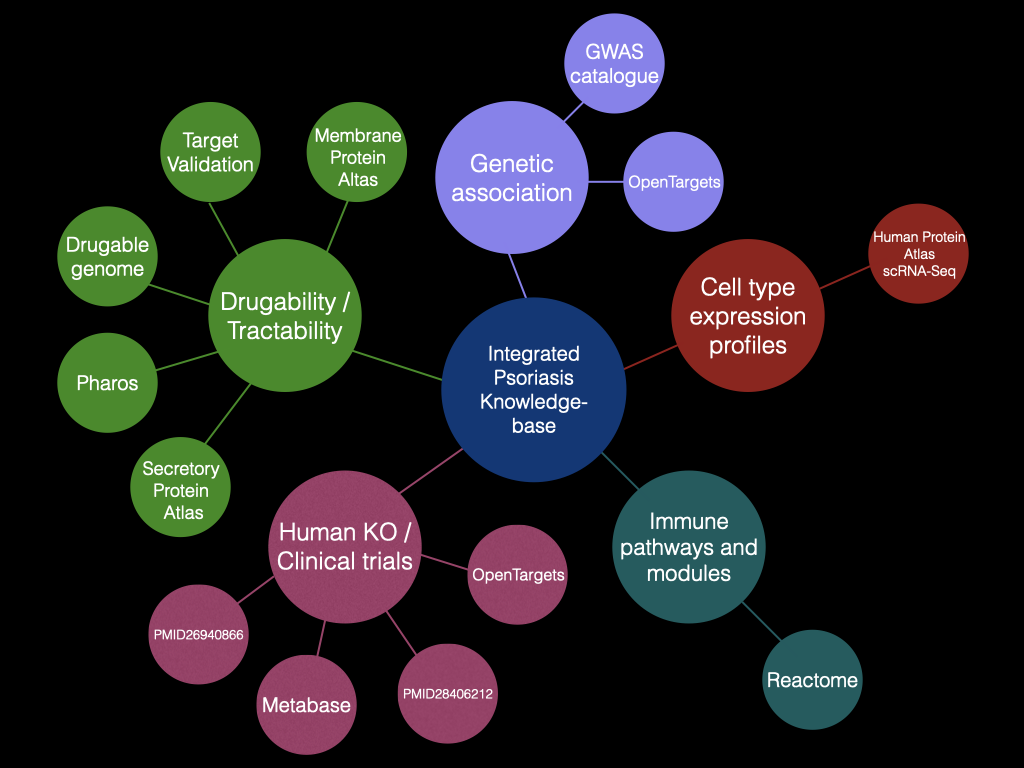


*Figure S5 - Graph-based representation of the Integrated Psoriasis Knowledge Base (IPKB). The central node represents the IPKB. The first order neighbors represent the category of data included in the IPKB. The second order neighbors represent the repositories and sources included in the IPKB.*


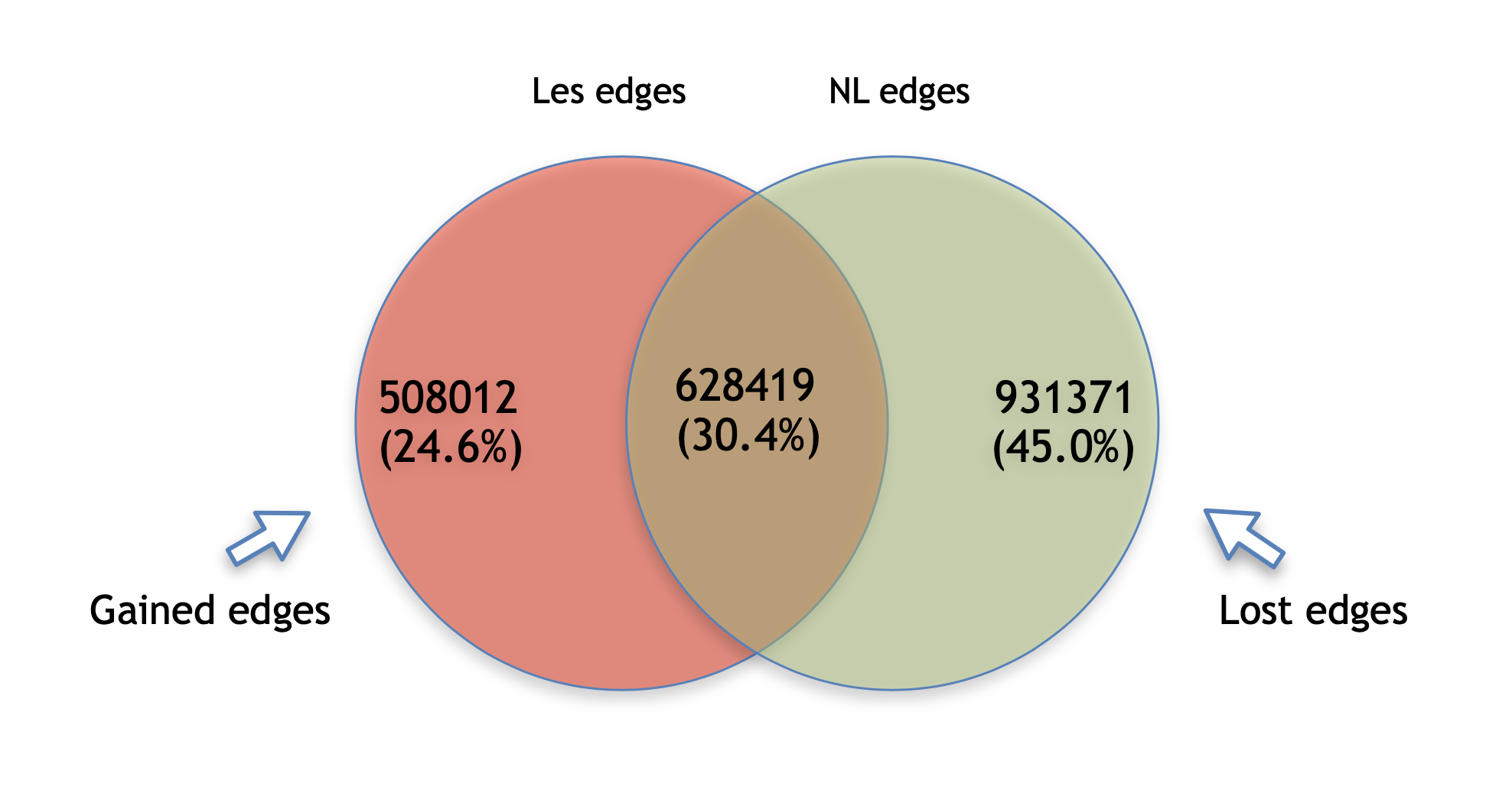


*Figure S6 - The figure shows a Venn diagram indicating: 1) the edges of the non lesional network which are not present in the lesional network (green circle), 2) edges not present in the non lesional network that are present in the lesional network (red circle) and 3) edges present in both of the networks (intersection between the red and the green circle).*

**
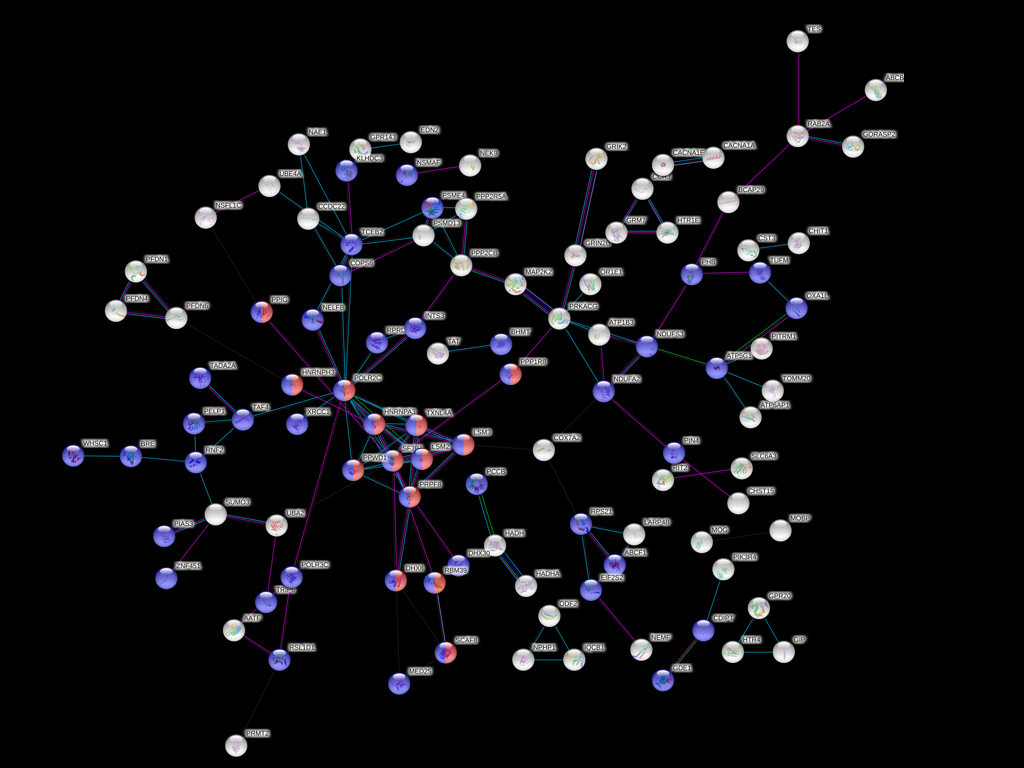
**

*Figure S7 - STRING protein-protein interaction network based on the selected bridge genes. Disconnected nodes were removed. Red color indicates the involvement of the gene in the RNA splicing process, while the blue color highlights the involvement of the genes in the cellular nitrogen compound metabolic process.*

*
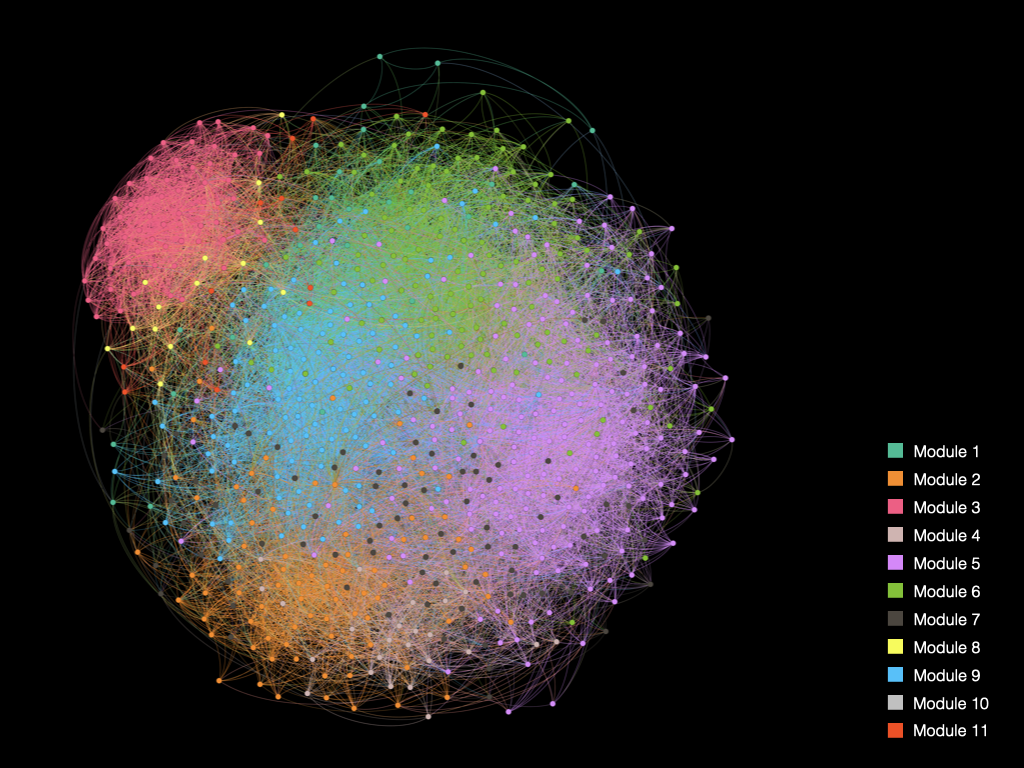
*

*Figure S8 - A) Gene co-expression network representing the lesional status of psoriatic skin. The different colors indicate the co-expression modules of size ≥ 10 genes. The network is a reduced representation of the actual network, in order to facilitate the visualisation.*

*
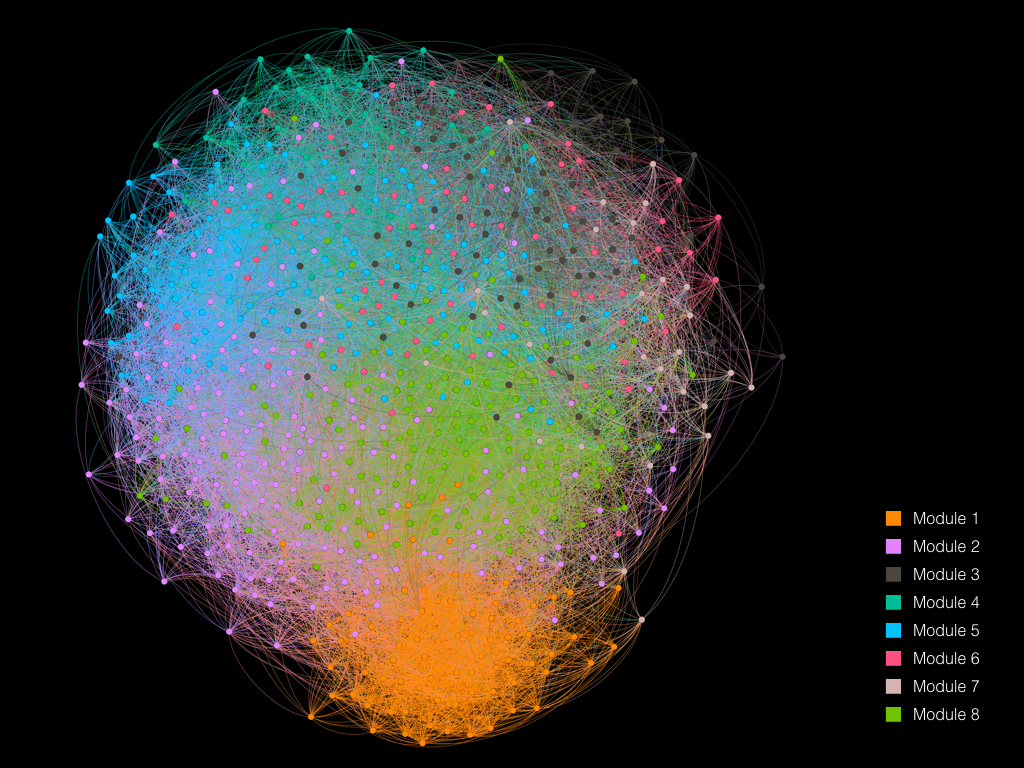
*

*Figure S9 - Gene co-expression network representing the non-lesional status of psoriatic skin. In both of the networks, the different colors indicate the co-expression modules of size ≥ 10 genes. Both of the shown networks are a reduced representation of the actual networks, in order to facilitate the visualisation.*

*
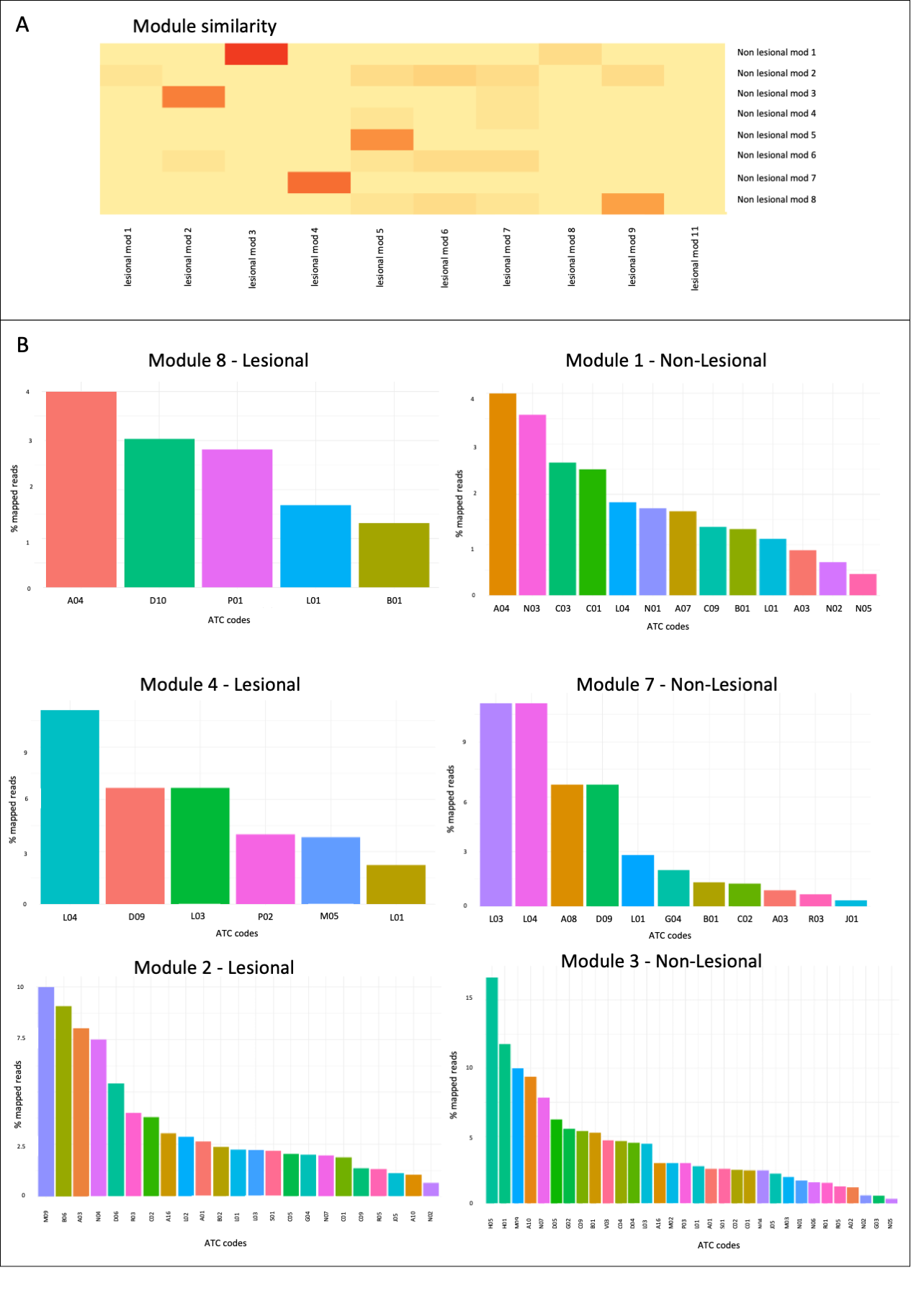
Figure S10 – Druggability analysis on differentially expressed genes on both the lesional and the non-lesional network models. Panel A shows modules similarities based on gene content between the two networks. Panel B shows the ATC categories and drugs targeting genes in modules with the highest similarity between the models.*
